# Supplementary material for: Population Genetic Structure of a Rare Butterfly in a Fragmented South Florida Ecosystem
Source: Insects. 2023 Mar 27;14(4):321. doi: 10.3390/insects14040321 (PMC10143422; doi:10.3390/insects14040321)
Supplement: Supplementary file 1 [file insects-14-00321-s001.zip › insects-2259240-supplementary.pdf]

Table S1: Microsatellite primer loci for *E. brunnea floridensis;* All F primers CAC-tag = CACGACGTTGTAAAAGG (=17bp)
All primer sequences listed 5′-3′

Locus Name	F*-sequence	R-sequence	Repeat motif	Repeat type	Size range (bp)	# alleles
Epbr 4864	TGAAAGTGAATTCGAAAATTG	ACAAACCCTCATGC GAGAAC	(AAAAG)^6	Pentanucleotide	212-294	6
Epbr 1052	ATTCACAGCGGACAGAGAT	CCACCTGTTACCGC CAAATA	(ACGC)^17	Tetranucleotide	214-242	4
Epbr 487	TCGGACCAGTATTCCTGAGA	CAGGAGGTCAGAC AGGCAAT	(AAAC)^23	Tetranucleotide	116-220	16
Epbr 5022	GCGTTTATGTGATGGTGCAT	TCAAACATGTGGTT GTCACG	(ATC)^14	Trinucleotide	98-200	4
Epbr 487b	TCGGACCAGTATTCCTGAGA	CAGGAGGTCAGAC AGGCAAT	(AAAC)^6	Tetranucleotide	336-352	4
